# Selective serotonin reuptake inhibitors to improve outcome in acute ischemic stroke: possible mechanisms and clinical evidence

**DOI:** 10.1002/brb3.373

**Published:** 2015-09-23

**Authors:** Timo Siepmann, Ana Isabel Penzlin, Jessica Kepplinger, Ben Min‐Woo Illigens, Kerstin Weidner, Heinz Reichmann, Kristian Barlinn

**Affiliations:** ^1^ Department of NeurologyUniversity Hospital Carl Gustav CarusTechnische Universität DresdenDresdenGermany; ^2^ Department of Psychotherapy and Psychosomatic MedicineUniversity Hospital Carl Gustav CarusTechnische Universität DresdenDresdenGermany; ^3^ Institute of Clinical PharmacologyUniversity Hospital Carl Gustav CarusTechnische Universität DresdenDresdenGermany; ^4^ Department of NeurologyBeth Israel Deaconess Medical CenterHarvard Medical SchoolBostonMassachusetts

**Keywords:** Acute ischemic stroke, fluoxetine, SSRI

## Abstract

**Background:**

Several clinical studies have indicated that selective serotonin reuptake inhibitors (SSRIs) administered in patients after acute ischemic stroke can improve clinical recovery independently of depression. Due to small sample sizes and heterogeneous study designs interpretability was limited in these studies. The mechanisms of action whereby SSRI might improve recovery from acute ischemic stroke are not fully elucidated.

**Methods:**

We searched MEDLINE using the PubMed interface to identify evidence of SSRI mediated improvement of recovery from acute ischemic stroke and reviewed the literature on the potential underlying mechanisms of action.

**Results:**

Among identified clinical studies, a well‐designed randomized, double‐blind, and placebo‐controlled study (FLAME ‐ fluoxetine for motor recovery after acute ischemic stroke) demonstrated improved recovery of motor function in stroke patients receiving fluoxetine. The positive effects of SSRIs on stroke recovery were further supported by a meta‐analysis of 52 trials in a total of 4060 participants published by the Cochrane collaboration. Based on animal models, the mechanisms whereby SSRIs might ameliorate functional and structural ischemic‐brain damage were suggested to include stimulation of neurogenesis with migration of newly generated cells toward ischemic‐brain regions, anti‐inflammatory neuroprotection, improved regulation of cerebral blood flow, and modulation of the adrenergic neurohormonal system. However, to date, it remains speculative if and to what degree these mechanisms convert into humans and randomized controlled trials in large populations of stroke patients comparing different SSRIs are still lacking.

**Conclusion:**

In addition to the need of comprehensive‐clinical evidence, further elucidation of the beneficial mechanisms whereby SSRIs may improve structural and functional recovery from ischemic‐brain damage is needed to form a basis for translation into clinical practice.

## Introduction

Despite the availability of safe and effective reperfusion therapies (i.e., intravenous thrombolysis and endovascular thrombectomy) acute ischemic stroke is still one of the leading causes of disability with presence of residual impairment in up to 75% of stroke survivors and annual costs amounting up to $74 billion in the United States alone (Hacke et al. 2008; Go et al. 2014; Berkhemer et al. 2015; Goyal et al. 2015). These epidemiological data suggest an urgent need for novel treatment strategies to improve poststroke recovery, particularly when viewed in conjunction with the ongoing demographic change toward population aging, not only in economically developed countries but also in less economically developed countries around the globe (Lutz et al. 2008).

Several clinical studies have indicated that treatment with selective serotonin reuptake inhibitors (SSRIs) might improve clinical recovery from acute ischemic stroke independently of depression but were limited by small sample sizes and heterogeneous designs (Dam et al. 1996; Zittel et al. 2008; Acler et al. 2009). However, in 2011 the discussion on the use of SSRIs to improve clinical outcome after stroke was reignited by positive results of a well‐designed randomized, double‐blind, placebo‐controlled study (FLAME ‐ fluoxetine for motor recovery after acute ischemic stroke) published by Chollet et al. (2011). This promising observation was further supported by the positive results of a synthesized analysis of 52 trials including secondary endpoint observations of functional and clinical outcomes published by the Cochrane collaboration (Mead et al. 2012). However, the mechanisms of action whereby SSRIs might improve recovery from stroke remain incompletely elucidated and a robust basis of prospective clinical and explorative research data to allow for the translation of SSRI‐induced stroke recovery into clinical practice is still lacking. This review summarizes the current literature on animal model‐based mechanistic hypotheses as well as clinical studies on the effects of SSRI treatment on clinical neurological outcomes and recovery from acute ischemic stroke.

## Search Methods and Study Selection Criteria

We performed a review which was not intended to be exhaustive. We searched MEDLINE using the PubMed interface. We included prospective‐controlled clinical trials and experimental animal studies conducted from 1994 to 2015. We only included clinical studies that assessed the effects of SSRI treatment on the following clinical neurological‐ and functional‐outcome parameters: disability (e.g. assessed using Barthel‐index), dependence (e.g. assessed using mRS‐modified Rankin scale), or neurological deficits (e.g. assessed using NIHSS‐National Institutes of Health Stroke Scale) at the end of treatment or at followup. We did not include studies that primarily investigated psychiatric or neuropsychological outcomes such as poststroke apathy or cognition. In this review of clinical studies, any agent classified as a SSRI was included (e.g. sertraline, citalopram, and fluoxetine). Additionally, we included experimental studies that aimed at assessing mechanisms of action of SSRI using animal models of acute ischemic stroke. We established a search strategy using the following MeSH terms and their combinations: “acute ischemic stroke,” “SSRI,” “selective serotonin reuptake inhibitors,” “dependency,” “disability,” “neurological deficits,” “functional outcome,” “clinical recovery,” “motor outcome,” “NIHSS,” “National Institutes of Health Stroke Scale,” “mRS,” “modified Rankin scale,” “Barthel‐index,” “fluoxetine,” “citalopram,” “fluvoxamine,” “escitalopram,” “sertraline,” and “paroxetine.” To search for mechanistic studies, we additionally used the MeSH terms “anti‐inflammation,” “neurogenesis,” “brain plasticity,” “auto‐regulation,” “cerebral blood flow,” “MCAO,” “middle cerebral artery occlusion,” and “animal model.” Additionally, we included preselected review papers and studies on the scientific background of this article for narrative‐introductory purposes.

## Possible Mechanisms of Action

**Figure 1:**
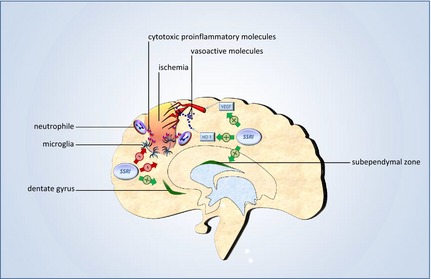
Possible mechanisms of action. The figure illustrates three possible mechanisms whereby SSRIs might improve structural‐brain tissue recovery from ischemia: stimulation of neurogenesis in the subependymal zone and hippocampal dentate gyrus, inhibition of microglia‐ and neutrophile‐induced inflammation mediated by cytotoxic inflammatory molecules, and improvement of cerebral vascular autoregulation (HO‐1, heme oxygenase‐1; VEGF, vascular endothelial growth factor).

A recent systematic review and meta‐analysis demonstrated that SSRIs improve infarct volume and neurobehavioral outcome in animal models of ischemic stroke (McCann et al. 2014). In animal studies, various beneficial mechanisms whereby SSRIs may improve structural and functional recovery from ischemic‐brain damage have been identified including, inter alia, enhancement of neuroplasticity, anti‐inflammation mediated neuroprotection, improvement of cerebral blood flow autoregulation, and modulation of the adrenergic neurohormonal system (Mead et al. 2012). These postulated mechanisms (illustrated in Fig. 1) might complement the primary psychopharmacological beneficial effects of reducing severity and frequency of poststroke depression and anxiety as well as improving sleep and alertness in the improvement of stroke recovery through SSRI treatment.

### Neuroplasticity

Neurotrophins constitute a class of proteins which contributes to both embryogenesis and organogenesis. Furthermore, neurotrophins regulate the ability of neural pathways and synapses to undergo morphological changes in response to environmental, behavioral, emotional, physical, and neurophysiological stimuli. This neural capacity of structural adaption is also referred to as neuroplasticity (Lang et al. 2004). Neurogenesis (the generation of new neurons from progenitor cells or stem cells) occurs in specific brain areas such as the ventricular subependyma and the subgranular area of the hippocampal dentate gyrus and was previously suggested to contribute to the recovery from ischemic stroke, complementing other cerebral repair mechanisms such as reorganization of the preexisting neural network and neurite sprouting (Taupin 2006). In animal studies, SSRIs were shown to enhance neurogenesis and expression of neurotrophins in the hippocampus (Schmidt and Duman 2007). This SSRI‐induced increase of intrahippocampal neurotrophin expression was linked to beneficial effects of antidepressant agents on behavior in mice (Santarelli et al. 2003). In fact, several animal studies have indicated that SSRI‐mediated stimulation of neurogenesis may contribute to structural and functional recovery from cerebral ischemia and that newly generated neurons may migrate from neurogenerating brain areas toward damaged regions (Liu et al. 1998; Gu et al. 2000; Jiang et al. 2001; Dempsey et al. 2003; Wiltrout et al. 2007). Interestingly, there is evidence that in patients with neurodegenerative disorders such as Alzheimer's disease, neurogenesis can occur within damaged brain regions further supporting the capability of this mechanism to specifically correspond to brain damage in a targeted fashion (Taupin 2006; Mu and Gage 2011). However, to date, it remains to be answered if and to what extent the SSRI‐mediated stimulation of neurogenesis after ischemic‐brain damage converts into human stroke patients.

### Neuroprotection

It is well established that inflammatory pathways contribute to late‐stage ischemic‐brain injury and thus deteriorate neurological outcome (Dirnagl et al. 1999; Kirino 2000). There is evidence from animal studies that reduction of inflammation through inhibition of microglia and neutrophil granulocytes by SSRIs might have the capacity to protect neurons when ischemia compromises structural integrity of brain cells (Dirnagl et al. 1999; Mead et al. 2012). In fact, rats undergoing middle cerebral artery occlusion (MCAO) had reduced infarct volume and less severe neurological deficits after intravenous application of fluoxetine compared to untreated control rats even when the drug was applied 9 hours postischemia (Lim et al. 2009). As discussed by the authors, it might be concluded that the anti‐inflammatory action of fluoxetine may contribute to neuroprotection in acute ischemic stroke by inhibiting late stages of postischemic inflammation and that their observation might be relevant to potential neuroprotective strategies in the treatment of human stroke patients.

### Cerebral blood flow regulation

In an animal model of focal‐ischemic infarction using mice undergoing cold laser‐induced ischemia in the medial frontal and somatosensory cortices, daily application of fluoxetine reduced extravasation and infarction size and improved cerebral blood flow regulation by normalizing the lower boundary of cerebral mean arterial pressure (Shin et al. 2009). Additionally, the authors observed an increase in the expression of heme oxygenase‐1 (HO‐1), which in turn leads to production of carbon monoxide and thus regulates vascular tone independently of nitric oxide synthase‐related pathways. Moreover this study noticed an increase in hypoxia‐inducible factor‐1alpha (HIF‐1alpha) in the ischemic‐brain region. This composite transcription factor activates various genes whose products are essential to oxygen homeostasis, including vascular endothelial growth factor (VEGF) (Ozaki et al. 1999). While these observations of SSRI‐induced improvement of cerebral blood flow regulation and the suggested underlying mechanisms appear plausible, their applicability to humans remains speculative.

### Modulation of the autonomic nervous system

In a study in rats, quantitative receptor autoradiography showed that treatment with citalopram or fluoxetine for 14 days induces upregulation of beta 1‐adrenergic receptors in the caudate–putamen and the somatosensory regions of the frontal cortex (Palvimaki et al. 1994). These results were replicated with multiple dosages of the study drugs, (citalopram: 2.5, 10 and 20 mg kg^−1^; fluoxetine 2.5, 10 and 20 mg kg^−1^) demonstrating reproducibility. However, the mechanism whereby beta 1‐adrenergic upregulation in ischemic‐brain regions might improve recovery remains to be answered. Additionally, another study in rats demonstrated that acute peripheral administration of SSRIs induces changes in autonomic cardiovascular function mediated by inhibition of sympathetic activity, presumably via a central mechanism (Tiradentes et al. 2014). However, it remains speculative whether SSRI‐induced central sympathoinhibition and consecutive changes in autonomic cardiovascular function (such as increase in heart rate variability or improved vasomotor reactivity) might contribute to post stroke recovery or have beneficial effects on prevention of recurrent cardiovascular events in humans.

### Cortical excitability

Motor cortex excitability is increased due to a decrease in motor intracortical inhibition in the affected hemisphere post stroke (Cramer et al. 1997; Chollet et al. 1991). In a randomized placebo controlled trial in twenty patients with unilateral stroke citalopram was shown to reduce motor cortex excitability measured using transcranial‐magnetic stimulation paralleled by improvement in neurological status assessed by NIHSS (Acler et al. 2009). A possible link between the observed decrease in cortical excitability and improved motor cortex recovery might be a beneficial effect of balanced bihemispheric motor cortex excitability on synaptic plasticity.

## Clinical Evidence

In a randomized, placebo‐controlled, three‐arm study published by Dam et al. (1996), 52 hemiplegic stroke patients were assigned to either placebo, tetracyclic antidepressant maprotiline (150 mg/day), or fluoxetine (20 mg/day) in addition to physical therapy for 3 months after stroke onset. The authors observed an improvement in walking and activities of daily living capacities assessed by the Barthel‐index in all study arms. Interestingly, greatest improvements were noticed in the fluoxetine group and lowest improvements were observed in the maprotiline group, indicating that fluoxetine might have facilitated clinical recovery from stroke. An alternative (or additional) explanation would be that maprotiline deteriorated stroke recovery. In addition to these alternative (or synergetic) conclusions, it is worth mentioning that, in this study, observed effects of drugs on clinical and functional‐outcome parameters were not related to their efficacy in improving depressive symptoms. Another study on eight acute ischemic‐stroke patients applied a placebo‐controlled, double‐blind, single‐dose, and crossover design to assess the effects of a single poststroke dose of citalopram on motor function (Zittel et al. 2008). The authors described that, in contrast to placebo, citalopram administration led to improvement of dexterity of the paretic hand assessed with the nine‐hole peg test. However, the study was limited by its small sample size.

In addition to functional‐ and clinical‐outcome parameters a study published by Acler et al. (2009) assessed motor area excitability in twenty SSRI‐treated acute ischemic‐stroke patients using transcranial‐magnetic stimulation (Acler et al. 2009). In a randomized, placebo‐controlled setting, the authors found that poststroke citalopram (10 mg/day) but not placebo decreased motor excitability and improved neurological deficits assessed using NIHSS. In fact, a placebo‐controlled, double‐blind, crossover study in eight patients with pure motor hemiparesis due to lacunar stroke revealed that, a single dose of fluoxetine led to hyperactivation in the ipsilesional primary motor cortex assessed with functional magnetic resonance imaging paralleling improvement in motor function of the affected side (Pariente et al. 2001).

The discussion on positive effects of SSRIs on recovery from acute ischemic stroke intensified after Chollet et al. published the results of the FLAME trial (Chollet et al. 2011; Cramer 2011; Majeed and Kamal 2011; Budhdeo and Deluca 2012). This randomized, double‐blind, and placebo‐controlled study enrolled 118 patients with acute ischemic stroke and randomly assigned these patients to either receive 20 mg fluoxetine daily (*n* = 59) or placebo (*n* = 59) poststroke in addition to physiotherapy for 3 months starting 5–10 days poststroke onset. The authors showed that in stroke patients with moderate to severe motor impairment, fluoxetine in combination with physiotherapy improved motor recovery after 3 months when compared to physiotherapy alone. Similar results were noticed in a double‐blind, randomized study in 83 acute ischemic‐stroke patients who were assigned to receive fluoxetine (*n* = 32), nortriptyline (*n* = 22), or placebo (*n* = 29) following acute ischemic stroke published in the same year as the FLAME trial (Mikami et al. 2011). In comparison with placebo, fluoxetine and nortriptylin led to a greater improvement in modified Rankin Scale scores.

This promising observation was further supported by the positive results of a metaanalysis of 52 trials on the effects of SSRIs on acute ischemic‐stroke patients (Mead et al. 2012). This synthesized data analysis in a total of 4060 study participants also included studies on poststroke depression treatment that assessed clinical and functional outcomes as secondary endpoints. The results of this analysis indicated that SSRI treatment is linked to improvement of dependence, disability, and neurological deficits after acute ischemic stroke. The observation of SSRI‐induced facilitation of functional and clinical recovery from acute ischemic stroke as well as external validity and reproducibility of this effect might be confirmed in the future by the results of currently ongoing multicentric studies (Mead; Mead et al. 2013; Kraglund et al. 2015; Mortensen and Andersen 2015).

In contrast to the aforementioned studies that focused on the effects of SSRIs administered after stroke onset, a previous study published by Mortensen et al. (2014) assessed the baseline stroke severity as well as the 30‐day mortality in acute ischemic‐stroke patients who were treated with SSRIs prior to stroke onset. This is an interesting approach as the possible mechanisms of SSRIs might be more effective in sustained treatment before, during, and after onset of stroke than in poststroke application when the onset of protective or repairing drug action succeeds the onset of ischemia‐induced brain damage. The hypothesis of superiority of prestroke SSRI over poststroke SSRI would also be consistent with a previous study in rats where chronic, but not acute, fluoxetine application increased hippocampal neurogenesis (Malberg et al. 2000). In their clinical registry‐based propensity score study, Mortensen et al. described that patients pretreated with SSRIs do not suffer from increased stroke severity but neither functional nor motor short‐term outcome were assessed in this investigation.

In summary, although the FLAME study and the Cochrane review showed promising results, the effect of SSRI in stroke recovery independent of depression has not yet been established and warrant further investigation.

## Relevance to Clinical Practice

While SSRIs are widely used to treat psychiatric complications after stroke such as poststroke depression, evidence of their capacity to improve stroke recovery in clinical practice is not sufficient to allow translation into clinical practice. In addition to efficacy, safety of SSRI use in poststroke patients needs to be considered in the clinical‐decision making process. The majority of studies on cerebrovascular risk were conducted in nonstroke populations and knowledge on recurrent events and mortality poststroke in SSRI‐treated stroke patients is limited (Mortensen and Andersen 2015). In individuals without a history of stroke SSRIs have been linked to increased risk of intracerebral hemorrhage but the absolute risk is low. The results from several ongoing trials might confirm safety and tolerability of SSRIs in poststroke patients as well as efficacy in enhancing stroke recovery (Mead et al. 2013; Kraglund et al. 2015; Mortensen and Andersen 2015). These awaited data might thus lead to a robust evidence based recommendation on whether SSRI should be used as standard pharmacotherapy in stroke recovery in the future.

## Perspective

Even though there is emerging evidence from clinical studies on the potential capacity of SSRIs to improve clinical and functional outcome from acute ischemic stroke, a lack of confirmatory trials in large‐study populations that also demonstrate generalizability and thus applicability in clinical practice remains. Additional questions that remain to be answered are to what extent animal study‐based postulated mechanisms mediating the beneficial effects of SSRIs in acute ischemic stroke convert into humans and whether sustained SSRI treatment started before the event might be superior over poststroke SSRI in improving recovery as recently suggested by a mono‐center observational study (Siepmann et al. 2015). The latter question appears particularly relevant given the fact that patients suffering from depression are at higher risk of a first‐ever stroke, and thus supporting the notion that SSRIs should become the first‐choice treatment for depression in the general population (Barlinn et al. 2014). In addition to the need of comprehensive‐clinical evidence, further elucidation of the beneficial mechanisms whereby SSRIs may improve structural and functional recovery from ischemic‐brain damage is needed to improve our understanding of this phenomenon and form a basis for translation into clinical practice.

## Conflict of Interest

The authors declare that there are no conflicts of interest in regard to the present work.
